# *Dryopteris juxtapostia* Root and Shoot: Determination of Phytochemicals; Antioxidant, Anti-Inflammatory, and Hepatoprotective Effects; and Toxicity Assessment

**DOI:** 10.3390/antiox11091670

**Published:** 2022-08-27

**Authors:** Abida Rani, Muhammad Uzair, Shehbaz Ali, Muhammad Qamar, Naveed Ahmad, Malik Waseem Abbas, Tuba Esatbeyoglu

**Affiliations:** 1 Department of Pharmaceutical Chemistry, Faculty of Pharmacy, Bahauddin Zakariya University, Multan 60800, Pakistan; 2 Department of Bioscience and Technology, Khwaja Fareed University of Engineering and Information Technology, Rahim Yar Khan 64200, Pakistan; 3 School of Environment and Safety Engineering, Jiangsu University, Zhenjiang 212013, China; 4 Institute of Food Science and Nutrition, Bahauddin Zakariya University, Multan 60800, Pakistan; 5 Multan College of Food & Nutrition Sciences, Multan Medical and Dental College, Multan 60000, Pakistan; 6 Institute of Chemical Sciences, Bahauddin Zakariya University, Multan 60800, Pakistan; 7 Institute of Food Science and Human Nutrition, Gottfried Wilhelm Leibniz University Hannover, Am Kleinen Felde 30, 30167 Hannover, Germany

**Keywords:** cytotoxicity, hepatoprotective effects, HeLa cancer, inflammation, mass spectrometry, oxidation, prostate cancer, phytochemical

## Abstract

An estimated 450 species of *Dryopteris* in the Dryoperidaceae family grow in Japan, North and South Korea, China, Pakistan, and Kashmir. This genus has been reported to have biological capabilities; however, research has been conducted on *Dryopteris juxtapostia*. Therefore, with the present study, we aimed to exploring the biological potential of *D. juxtapostia* root and shoot extracts. We extracted dichloromethane and methanol separately from the roots and shoots of *D. juxtapostia*. Antioxidant activity was determined using DPPH, FRAP, and H_2_O_2_ assays, and anti-inflammatory activities were evaluated using both in vitro (antiurease activity) and in vivo (carrageenan- and formaldehyde-induced paw edema) studies. Toxicity was evaluated by adopting a brine shrimp lethality assay followed by determination of cytotoxic activity using an MTT assay. Hepatoprotective effects of active crude extracts were examined in rats. Activity-bearing compounds were tentatively identified using LC-ESI-MS/MS analysis. Results suggested that *D. juxtapostia* root dichloromethane extract exhibited better antioxidant (DPPH, IC_50_ of 42.0 µg/mL; FRAP, 46.2 mmol/g; H_2_O_2_, 71% inhibition), anti-inflammatory (urease inhibition, 56.7% at 50 µg/mL; carrageenan-induced edema inhibition, 61.7% at 200 µg/mL; formaldehyde-induced edema inhibition, 67.3% at 200 µg/mL), brine shrimp % mortality (100% at 1000 µg/mL), and cytotoxic (HeLa cancer, IC_50_ of 17.1 µg/mL; prostate cancer (PC3), IC_50_ of 45.2 µg/mL) effects than *D. juxtapostia* root methanol extract. *D. juxtapostia* shoot dichloromethane and methanol extracts exhibited non-influential activity in all biological assays and were not selected for hepatoprotective study. *D. juxtapostia* root methanol extract showed improvement in hepatic cell structure and low cellular infiltration but, in contrast the dichloromethane extract, did not show any significant improvement in hepatocyte morphology, cellular infiltration, or necrosis of hepatocytes in comparison to the positive control, i.e., paracetamol. LC-ESI-MS/MS analysis showed the presence of albaspidin PP, 3-methylbutyryl-phloroglucinol, flavaspidic acid AB and BB, filixic acid ABA and ABB, tris-desaspidin BBB, tris-paraaspidin BBB, tetra-flavaspidic BBBB, tetra-albaspidin BBBB, and kaempferol-3-*O*-glucoside in the dichloromethane extract, whereas kaempferol, catechin, epicatechin, quinic acid, liquitrigenin, and quercetin 7-*O*-galactoside in were detected in the methanol extract, along with all the compounds detected in the dichloromethane extract. Hence, *D. juxtapostia* is safe, alongside other species of this genus, although detailed safety assessment of each isolated compound is obligatory during drug discovery.

## 1. Introduction

Generation of free radicals in living systems is associated with intrinsic (stress) and extrinsic (alcohol, smoking, and radiation) factors, whereas antioxidant mechanisms help to neutralizing the negative impacts induced by oxidative stress [1]. Oxidative stress is a condition whereby imbalance occurs between reactive oxygen species (ROS) and the body’s antioxidant system. Undue generation of ROS disrupts the normal functioning of important organs and can lead to the onset of many ailments, including inflammation, cancer, polygenic disorders, diabetes, and aging [2,3,4].

Pakistan, regardless of being an agricultural territory, is home to various ecological zones with many indigenous medicinal plant species [5,6]. However, limited research has been conducted to evaluate their pharmaceutical prominence due to the phytochemical potential of secondary metabolites [7]. Of 5700 reported medicinal plant species, Pakistan possesses 500-600 species, with only a few having been probed for biochemical assessment [8]. The *Dryopteris* genus of the Dryoperidaceae family of the North Temperate Zone comprises more than 450 species grown in Japan, North and South Korea, China, Pakistan, and Kashmir [9]. *Dryopteris ramose* and *Dryopteris cochleata* extracts, i.e., species from the aforementioned genus, have been reported to exhibit antioxidant activity [10,11]. *Dryopteris chrysocoma*, *Dryopteris blanfordii*, and *Dryopteris* *crassirhizoma* were reported to exhibit anti-inflammatory activities [12,13,14]. In other studies, *Dryopteris fragrans* and *Dryopteris crassirhizoma* extracts were found to possess anticancer potential [15,16]. However, *Dryopteris juxtapostia* extracts have not been explored for biological potential to date. Therefore, the aim of the present study is to explore phytochemical, radical-scavenging, anti-inflammatory, cytotoxic, and hepatoprotective potential of *Dryopteris juxtapostia* root and shoot extracts.

## 2. Materials and Methods

### 2.1. Plant Material and Its Preparation

*Dryopteris juxtapostia* (DJ) roots and shoot were collected from the Sawat area (Tehsil Matta upper swat. KPK., Village, Shukhdara, Biha, Charrma, Fazal Banda), Pakistan, and identified and authenticated at the Institute of Pure and Applied Biology, Bahauddin Zakariya University, Multan, Pakistan. The plant was assigned voucher no. tro-26609785. For the purpose of effective extraction, whole DJ root and shoot material was shade-dried for 15 days. Then, dried plant material was ground in a blender and weighed. Extraction was performed with dichloromethane and methanol in an orbital shaker in the dark for 48 h. The process was repeated three times, and the solvent was evaporated using a rotary evaporator (Heidolph, Schwabach, Germany) in to obtain semisolid plant material.

### 2.2. Quantification of Total Phenolic and Flavonoid Contents

Total phenolic contents were determined by Folin–Ciocalteu (FC) colorimetric assay using gallic acid as standard [17]. Absorbance was recorded at 765 nm using a spectrophotometer (UV-Vis 3000 ORI, Reinbeker, Germany), and values were recorded in triplicate using ethanol as a blank. Total flavonoid contents were determined using an AlCl_3_ assay [18]. Sample absorbance was read at 510 nm using a spectrophotometer (UV-Vis 3000 ORI). A quercetin standard curve was plotted; sample results are expressed as mg quercetin equivalents per gram (mg QE/g) of the dried weight.

### 2.3. Antioxidant Activity

DJ dichloromethane and methanol crude extracts of roots and shoots were evaluated for antioxidant activity using three assays i.e., DPPH, H_2_O_2_, and FRAP assays, as adopted by Qamar et al. (2021) [19]. Samples were prepared as 1 g/20 mL for all three assays. In the DPPH and H_2_O_2_ assays, distilled water was used as a blank, with quercetin (125 µg/mL) as standard. The findings are reported as percent inhibition according to the following equation:

%Inhibition = [(absorbance of control − absorbance of sample/standard) ÷ absorbance of control] × 100

For the FRAP assay, ferrous sulphate was used for calibration. Results are expressed as Fe mmol/g.

### 2.4. Anti-Inflammatory Activity

#### 2.4.1. Urease Inhibition Assay (In Vitro)

Weatherburn’s indophenol method was used to evaluate the urease activity by determining the ammonia production in the reaction mixture [20]. The reaction mixture comprising 25 µL jack bean urease enzyme, 55 µL buffer (100 mM urea), and 5 µL test compounds (0.5 mM) was incubated for 15 min in a 96-well plate at 30 °C. Urease activity was assessed by Weatherburn’s method by measuring ammonia production using indophenol. In brief, 70 µL alkali (0.1% active chloride (NaOCl) and 0.5% NaOH *w/v*) and 45 µL phenol reagents (0.005% *w/v* sodium nitroprusside and 1% *w/v* phenol) were added to each well. After 50 min, the increase in absorbance was measured at 630 nm with a microplate reader (Molecular Device, Ramsey, NJ, USA). The reaction was performed in a triplicate run, with pH 6.8 and a final volume of 200 µL. Absorbance readings were processed with Max Pro software (Molecular Device, USA), and % inhibition was calculated using following equation:

$$\mathrm{Percentage}\;\mathrm{inhibitions}\;\left(\%\right)=100-\left(\;\frac{\mathrm{OD}\;t}{\mathrm{OD}\;c}\;\right)\times 100$$

where OD t is optical density of the test well, and OD c is the optical density of the control. Thiourea was used as the standard urease inhibitor in this study.

#### 2.4.2. Carrageenan- and Formaldehyde-Induced Paw Oedema (In Vivo)

A carrageenan-induced paw inflammation assay was employed to assess the pain-relieving capabilities of DJ dichloromethane and methanol (root and shoot) extracts in rats according to Morris (2003) [21], with some modifications. The study was performed by adopting the parameters mentioned in the guidelines of the National Research Council [22] (NRC, 1996, Washington, DC, USA). The study was also approved by the departmental Committee pf Animal Care at BZU, Pakistan (approval number ACC-10-2019). Rats were divided into six groups (n = 5); animals in group 1 were provided with normal saline and designated the control group. Animals in group 2 were given standard indomethacin at a dose of 100 mg/kg body weight (b.w.) and designated the positive control. Rats in groups 3 and 4 were fed with the dichloromethane extract (200 mg/kg) of *D. juxtapostia* roots and shoots, respectively. Rats in groups 5 and 6 were fed with the methanol extract (200 mg/kg) of *D. juxtapostia* roots and shoots, respectively. One half hour after extract administration, the animals were injected with carrageenan into the plantar aponeurosis surface of the right hind paw. Any change in paw linear circumference was noted after 0, 1, 2, and 3 h using a plethysmometer (UGO-BASILE 7140, Comerio, Italy). An increase in paw circumference was taken as indicator of inflammation.

Likewise, a formaldehyde-induced hind-paw edema assay was used to examine the anti-inflammatory potential of DJ dichloromethane and methanol (root and shoot) extracts in mice, adopting the method of Brownlee with minor changes [23]. We divided animal into a total of six study groups; details are the same as those mentioned above for the carrageenan-induced paw inflammation assay. One half hour after extract administration, formaldehyde (100 μL, 4%) was injected into the plantar aponeurosis of each mouse’s right paw, and changes in paw circumference were recorded after 0, 3, 6, 12, and 24 h.

### 2.5. Brine Shrimp Lethality Assay

The method described by Meyer et al. (1982) [24] was used to perform a brine shrimp lethality assay. Commercial salt was dissolved in distilled water to prepare artificial seawater in a rectangular plastic tray (22 × 32 cm) in the dark. Fifty milligrams of shrimp eggs (*Artemia salina*) obtained from Husein Ebrahim Jamal Research Institute of Chemistry (HEJ, Karachi, Pakistan) was scattered into the artificial seawater. Incubation lasted 48 h at 37 °C. Pasteur pipettes were used to collect hatched larvae. Dichloromethane and methanol extracts from roots and shoots of DJ were prepared at concentrations of 10, 100, and 1000 µg/mL. Samples with varying strengths were separately transferred to clean vials. Each incubation vial contained 1 mL artificial seawater (to a final volume of 5 mL) and 30 shrimp with pH 7.4 adjusted using 1N NaOH and incubated for 24 h at 26 °C. The shrimp survival rate was quantified in each vial, including the positive control (i.e., etoposide).

### 2.6. Cytotoxic Activity

To assess the cytotoxic potential of DJ dichloromethane and methanol extracts (root and shoot), we adopted the method described by Mosmann et al. (1983) [25]. Experimental samples of varying strengths (0.5–200 μg/mL) were prepared in 100 μL dimethylsulphoxide (1% *v/v*) in 96-well microtiter plates. After incubating the microtiter plates (37 °C, 48 h), 50 μL of the MTT solution (5 mg/mL) was added to each well. A microplate reader was used to check the reduction in MTT after a second incubation (37 °C for 4 h) by recording the absorbance at 570 nm. The untreated cells were used as a control against which to measure the effect of experimental extracts on the cell viability. The percent inhibition exhibited on the cell cultures by the test samples was computed using the following equation:

Survival (%) = (At − Ab)/(Ac − Ab) × 100

where At, Ab, and Ac indicate the sample, blank (complete media without cells), and control absorbance, respectively.

Cell inhibition (%) = 100 − cell survival (%)

### 2.7. Hepatoprotective Studies

The active crude extracts of DJ roots and parts were subjected to hepatoprotective analysis following the OECD 423 (Organization for Economic Co-operation and Development) guidelines [26]. Preset parameters and guidelines of the National Research Council (1996, Washington, USA) were also considered. The institutional ethical committee of Bahauddin Zakariya University (BZU) Multan Pakistan approved the animal study under the title “Study of hepatoprotective potential of *Dryopteris juxtapostia*”. The regimen presented in Table 1 was used to orally administer DJ plant extracts to the groups for ten days, but only the most biologically active extracts were considered for this analysis. All animals were treated according to the regimen presented in Table 2. After last dose, retro-orbital plexus blood was collected. Blood of animals in each treatment group was saved for lipid and protein analysis. Furthermore, serum samples were allowed to clot for 60–70 min at ambient room temperature to test for biochemical liver function markers, followed by centrifugation (2500 rpm at 30 °C) for 15–20 min.

#### 2.7.1. Assessment of Liver Functions

Serum glutamic-pyruvic transaminase (SGPT)/alanine aminotransferase (ALT), serum glutamic-oxaloacetic transaminase (SGOT)/aspartate aminotransferase (AST), alkaline phosphate, total bilirubin, total protein, and lipid profile were analyzed and quantified in each the serum from each group. The activity of serum transaminases (SGPT and SGOT) and blood lipid profile were examined using the Rietman and Frankel method [27]. Total protein was evaluated according to the Lowry procedure [13]. Total bilirubin (TB) and alkaline phosphate (ALP) were estimated using the methodologies described by Keiding et al. (1974) [28] and Tietz et al. (1983) [29], respectively.

#### 2.7.2. Histopathology of Liver

The liver of all experimental animals (rats) were excised and washed with normal saline. The cleansed liver tissues were separately preserved in 10% formalin solution (neutral) in air-tight, labelled jars. After eight days, the tissues were dehydrated using ethanol solution. The tissues were dried, embedded in paraffin, and sliced into 5 µm length segments. The liver sections were placed on a marked slide and dyed (hematoxylin–eosin (H & E) 400X). The prepared labelled slides were then observed under a photomicroscope (Olympus-CX23 Upright, Japan) for vacuolar degeneration, cellular infiltration, and necrosis of hepatocytes.

### 2.8. LC-ESI-MS/MS Analysis of Active Crude Extracts

Crude extracts exhibiting biological potential and outlined hepatoprotective effects were further subjected to mass spectrometry analysis using LC-ESI-MS/MS (Thermo Electron Corporation, Waltham, MA, USA) with the aim of tentative identification of activity-bearing compounds. Detection was carried out by adopting direct-injection-mode ESI (electron spray ionization) in both negative and positive modes. Range of mass, temperature of capillaries, and sample flow rate were maintained at *m/z* 50 to 1000, 280 °C, and 8 µL/min, respectively. Collision-induced energy generated during MS/MS analysis depended upon the nature/type of the parent molecular ion subjected to 10 to 45 eV. Furthermore, in order to ensure sufficient ionization and ion transfer, every compound was optimized for MS parameters. Similarly, for every analyte, the source parameters were unchanged but parent, whereas daughter signals were optimized either by analyte infusion or manually. Moreover, online mass data banks, software, and previously published literature were used for compound identification (www.chemspider.com, accessed on 12 December 2021).

### 2.9. Statistical Analysis

Study data are expressed as the mean (SEM) of three measurements. ANOVA was used to compare the differences between the control and treatment groups, and Dunnett’s test was run using GraphPad Prism (Graph Pad Software V8, San Diego, CA, USA).

## 3. Results

### 3.1. Phytochemical Constituents and Antioxidant Activity of Dryopteris juxtapostia (DJ) Crude Extracts

**Figure 1:**
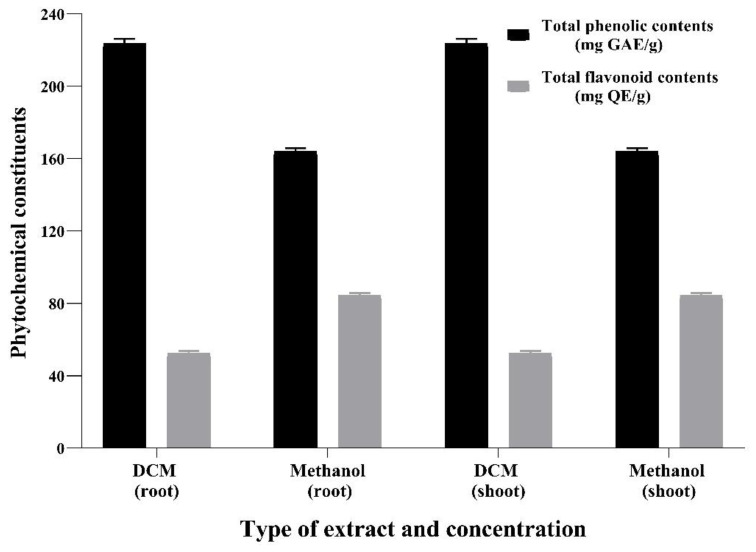
Total phenolic and flavonoid contents of *Dryopteris juxtapostia* root and shoot crude extracts. Values are presented as means ± S.D. of three measurements.

The quantitative investigation recorded the maximum total phenolic contents in 100% DCM extract of *D. juxtapostia* roots, root methanol extract, shoot DCM extract, and shoot methanol extract as 222 ± 0.41 mg GAE/g, 163 ± 0.2 mg GAE/g, 109 ± 0.41 mg GAE/g, and 91.4 ± 0.2 mg GAE/g, respectively (Figure 1). In contrast, total flavonoid contents recorded in methanol extracts of *D. juxtapostia* roots and shoots, i.e., 83.7 ± 0.1 mg QE/g and 43.8 ± 0.3 mg QE/g, respectively, were higher compared to those of DCM root and shoot extracts, i.e., 51 ± 0.2 mg QE/g and 13.2 ± 0.5 mg QE/g, respectively.

**Figure 2:**
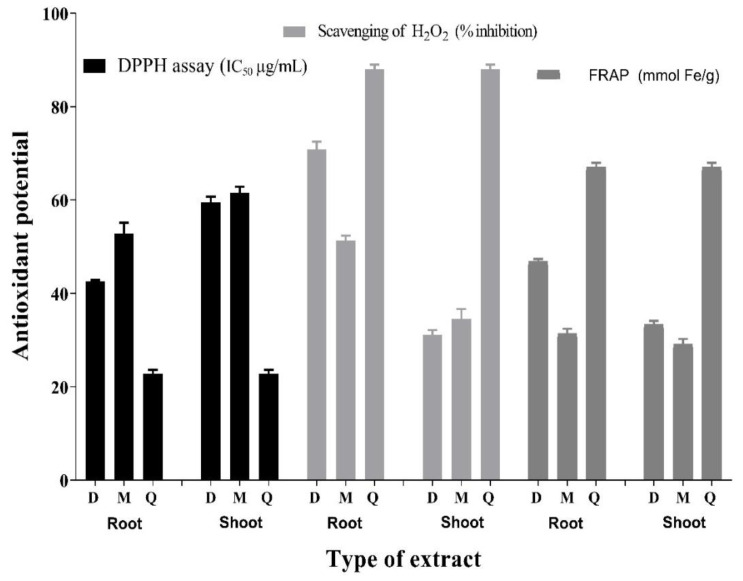
Antioxidant activity of *Dryopteris juxtapostia* root and shoot crude extracts. D, 100% dichloromethane extract; M, 100% methanol extract; Q, quercetin (standard). Values are presented as means ± S.D. of three measurements.

The radical-scavenging ability of various *D. juxtapostia* crude extracts (shoot DCM extract, shoot methanol extract, root dichloromethane extract, and root methanol extract) was evaluated using various antioxidant assays, such as stable radical assay (DPPH), reducing assay (FRAP), and hydrogen peroxide (H_2_O_2_) inhibition assay. As shown in Figure 2, among all extracts, *D. juxtapostia* root DCM extract exhibited the lowest IC_50_ of 42.0 µg/mL against stable free radicals (DPPH), followed by root methanol extract, with an IC_50_ of 54.0 µg/mL. In contrast, in the present study, moderate activity was shown by *D. juxtapostia* shoot DCM and methanol extracts, with IC_50_ values of 59.0 µg/mL and 61.4 µg/mL, respectively. Quercetin was used as a standard antioxidant compound and exhibited remarkable activity, with an IC_50_ of 22.3 µg/mL.

*D. juxtapostia* root DCM extract demonstrated a higher reducing potential of 46.2 mmol/g, followed by shoot dichloromethane extract, root methanol extract, and shoot methanol extract, with a reducing potential of 31.1 mmol/g, 34.6 mmol/g, and 29.4 mmol/g, respectively. Quercetin was observed to have the highest reducing potential of 66.0 mmol/g.

In the hydrogen peroxide inhibition assay, *D. juxtapostia* root dichloromethane extract demonstrated 71.0% inhibition against delineated prominent activity relative to the standard quercetin (87.0% inhibition) and in contrast to root methanol extract (51.0% inhibition), shoot dichloromethane extract (32.1% inhibition), and methanol extract (34.2% inhibition).

### 3.2. In Vitro Anti-Inflammatory Activity

**Figure 3:**
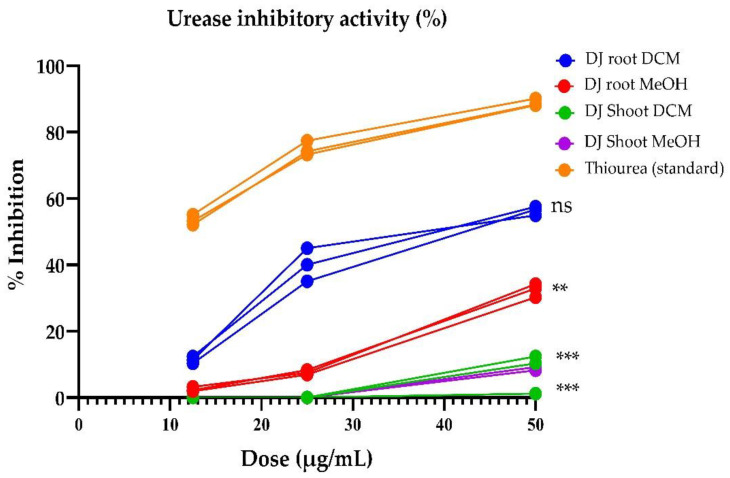
Urease inhibition activity (%) of various *D. juxtapostia* crude extracts. DCM, dichloromethane; DJ, *Dryopteris juxtapostia*; MeOH, methanol; ns, non-significant. Values are presented as means ± S.D. of three readings. ** *p* < 0.01. *** *p* < 0.001.

*D. juxtapostia* crude extracts were evaluated for possible antiurease activity at varying concentrations, i.e., 12.5, 25, and 50 µg/mL, using thiourea as a standard anti-inflammatory drug. The results presented in Figure 3 illustrate that *D. juxtapostia* root dichloromethane extract exhibited urease inhibition activity of 56.7% at 50 µg/mL, followed by root methanol extract, with moderate urease inhibition activity of 32.9% at 50 µg/mL. Similarly, the standard drug, i.e., thiourea, exhibited potent inhibition of 88.9% at 50 µg/mL. In contrast, *D. juxtapostia* shoot dichloromethane and methanol extracts evinced non-influential antiurease activity at all concentrations. The anti-inflammatory activity of *D. juxtapostia* root dichloromethane and methanol extracts was found to be consistent with total phenolic contents and antioxidant activity. Statistically, activity outlined by DJ root DCM extract was comparable to that of standard thiourea, with a non-significant difference (ns) observed between their activities, whereas the activity of DJ root MeOH (*p* < 0.01), DJ shoot DCM (*p* < 0.001), and DJ shoot MeOH *p* < 0.001) extracts was significantly lower when compared to standard thiourea (Figure 3).

### 3.3. In Vivo Anti-Inflammatory Activity

In the present study, the experimental crude extracts, including *D. juxtapostia* root dichloromethane and methanol extracts and *D. juxtapostia* shoot dichloromethane and methanol extracts were evaluated for possible in vivo pain-alleviating properties induced by carrageenan and formaldehyde at various concentrations, i.e., 50, 100, and 200 mg/kg (Table 2).

DJ root dichloromethane extract showed an antiedematous effect in a dose-dependent manner, with a maximum inhibition of 61.7% (*p* < 0.001) at 200 mg/kg after 3 h, in contrast to the control (normal saline). This is comparable to the anti-inflammatory effects of the standard anti-inflammatory drug indomethacin at a dose of 100 mg/kg, which altered inflammation by as much as 77.6% (*p* < 0.0001). In contrast, *D. juxtapostia* root methanol extract evinced moderate inhibition (43.9% at 200 mg/kg), whereas *D. juxtapostia* shoot extracts exhibited non-substantial activity when compared to the control. The in vivo anti-inflammatory properties of *D. juxtapostia* are in line with its in vitro anti-inflammatory, antioxidant, and phytochemical potential.

Akin to the previous model of inflammation, *D. juxtapostia* root dichloromethane extract, when administrated at a rate of 200 mg/kg, evinced inhibition of 67.3% (*p* < 0.001) after 24 h against formaldehyde-induced pain behavior in contrast to the control, i.e., normal saline. The activity was parallel to the anti-inflammatory effects of the standard anti-inflammatory drug indomethacin at a dose of 100 mg/kg, which altered inflammation by as much as 86.3% (*p* < 0.0001). Moreover, *D. juxtapostia* root methanol extract showed moderate inhibition of 45.1% at 200 mg/kg, whereas both extracts of the shoot portion, i.e., dichloromethane and methanol, exhibited non-substantial activity against formaldehyde-intoxicated pain behavior (Table 3).

### 3.4. Brine Shrimp Lethality Assay

Brine shrimp lethality assay is an imperative method for determining the preliminary cytotoxicity of experimental plant extracts and other substances based on their ability to kill laboratory-cultured larvae (nauplii). Such an assay is easy to use, inexpensive, and requires only a small amount of test material. In the present study, the cytotoxicity of various crude extracts of *D. juxtapostia* were evaluated at varying concentrations, i.e., 10, 100, and 1000 µg/mL, to compute % mortality. Dichloromethane extracts were observed to be more cytotoxic in comparison to methanol extracts in a dose-dependent manner (Table 4). In brief, *D. juxtapostia* root dichloromethane extract was found to be the most lethal of all investigated extracts, with 100%, 76%, and 10% mortality at 1000 µg/mL, 100 µg/mL, and 10 µg/mL, respectively, followed by standard etoposide (70% mortality at 10 µg/mL), *D. juxtapostia* shoot dichloromethane extract, *D. juxtapostia* root methanol extract, and *D. juxtapostia* shoot methanol extract. These findings revealed that *D. juxtapostia* root dichloromethane extract may contain some compounds that exert cytotoxic effects on certain cancer cells.

### 3.5. Cytotoxic Activity of Various D. juxtapostia Crude Extracts Using MTT Assay

*D.juxtapostia* root dichloromethane extract, root methanol extract, shoot dichloromethane extract, and shoot methanol extract were evaluated for possible anticancer potential using doxorobicin as a standard anticancer drug by MTT assay (Table 5). The MTT (3-[4,5-dimethylthiazol-2-yl]-2,5 diphenyl tetrazolium bromide) assay is based on the conversion of MTT into formazan crystals by living cells, which determines mitochondrial activity. Because for most cell populations, the total mitochondrial activity is related to the number of viable cells, this assay is broadly used to measure the in vitro cytotoxic effects of drugs on cell lines or primary patient cells.

*D.juxtapostia* root dichloromethane extract was not only found to anticipate a reduction in oxidative stress induced by DPPH, FRAP, and H_2_O_2_ but also yielded significant inhibition in cancer progression among both investigated cancer cell lines, i.e., HeLa human cervical and prostate cancer cell lines (PC3), with an IC_50_ of 17.1 µg/mL and 45.2 µg/mL, respectively. Moreover, *D. juxtapostia* root methanol extract demonstrated prominent inhibitory activity against the Hela cancer cell line, with an IC_50_ of 36.9 µg/mL, and moderate activity against human prostate cancer cell lines, with an IC_50_ of 98.3 µg/mL. The standard anticancer drug doxorubicin exhibited potent inhibition against both cancer cell lines, with IC_50_ values of 0.90 µg/mL (HeLa human cervical cancer cell line) and 1.90 µg/mL (PC3).

### 3.6. Hepatoprotective Activity

**Figure 4:**
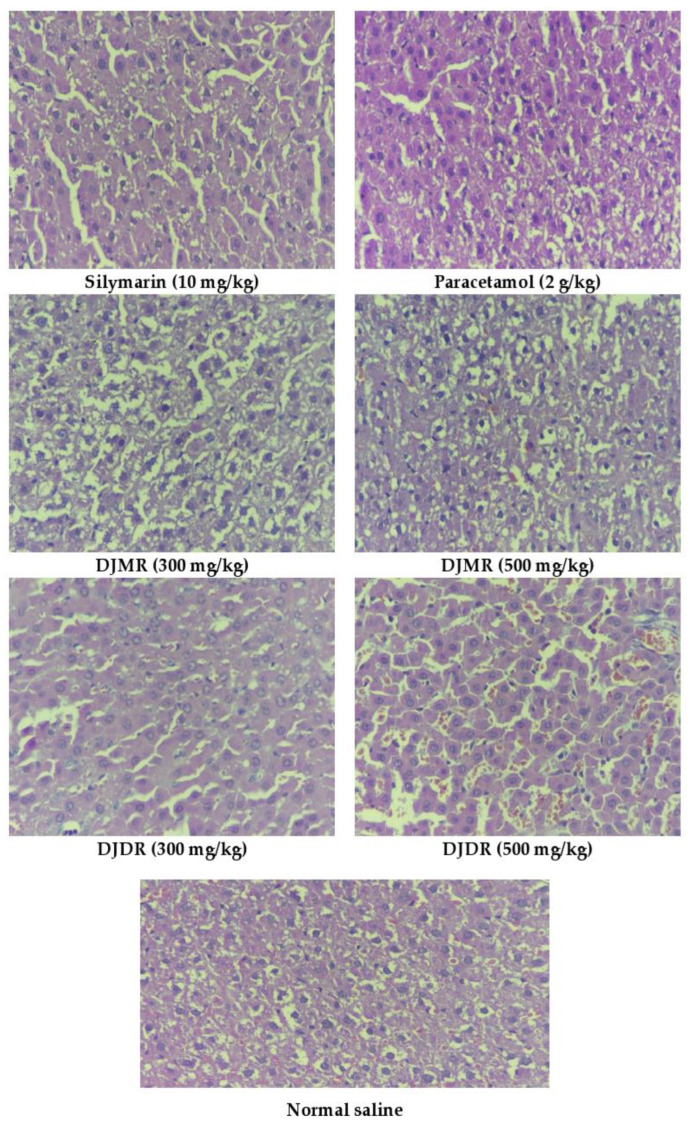
Histopathological analysis of liver. DJDR, *Dryopteris juxtapostia* methanol root; DJMR, *Dryopteris juxtapostia* dichloromethane root.

Histopathology results revealed normal hepatocytes with no inflammatory changes in the group given silymarin (group 3). However, the group treated with paracetamol (group 2) showed fatty changes, vacuolar degeneration, cellular infiltration, and necrosis of hepatocytes. Groups given *D. juxtapostia* root methanol extract at 300 mg/kg (group 6) and 500 mg/kg (group 7) showed improvement in cell structure, and low cellular infiltration was observed at a higher dose as compared to a lower dose. Groups 4 and 5 given *D. juxtapostia* root dichloromethane extract at doses of 300 mg/kg and 500 mg/kg, respectively, did not show any notable improvement in hepatocyte morphology, cellular infiltration, or necrosis of hepatocytes, as shown in Figure 4. As shown in Table 6, *D. juxtapostia* root methanol extract was found to be more effective in a liver function test, as well as total protein, and total lipid profile tests, in a dose-dependent manner as compared to *D. juxtapostia* root dichloromethane extract.

### 3.7. Mass Spectrometry Analysis of Various Extracts

*D. juxtapostia* root dichloromethane and methanol extracts showing notable biological activities were subjected to mass spectrometry analysis (ESI-MS/MS) to identify (tentative) compounds by comparing the mass spectra and their fragments with mass banks and previously published literature. In detail, albaspidin PP, 3-methylbutyryl-phloroglucinol, flavaspidic acid AB, flavaspidic acid BB, filixic acid ABA, filixic acid ABB, tris-desaspidin BBB, tris-paraaspidin BBB, tetra-flavaspidic BBBB, tetra-albaspidin BBBB, and kaempferol-3-*O*-glucoside were detected in DCM extract. All the aforementioned compounds were also detected in methanol extract, along with kaempferol, catechin, epicatechin, quinic acid, liquitrigenin, and quercetin 7-*O*-galactoside (Table 7). Compound (**A**) was previously identified in another species of the same genus called *Dryopteris crassirhizoma* [30]. Compounds (**B**–**I**) were also identified in the same genus in a species called *Dryopteris Adanson* [31]. Compounds (**J**–**M**) were identified according to recent literature reports [19,32,33].

## 4. Discussion

*D. juxtapostia* root dichloromethane and methanol extracts were found to have higher phenolic and flavonoid contents, respectively, as compared to shoot extracts (Figure 1). The identical potential of bioactive metabolites was reported in an previous study by Baloch et al. (2019) [34], wherein dichloromethane and methanol extracts of *Dryopteris ramose* belonging to the same genus as our experimental plant exhibited notable phenolic (184.2–199.2 mg GAE/g) and flavonoid (50.13–73.02 mg rutin equivalent (RE)/g) contents, supporting the findings of the present investigation. Another study revealed that *Dryopteris ramose* was high in total flavonoid contents using various solvents, including ethyl acetate extract (45.28 μg QE/mg), methanol extract (36.94 μg QE/mg), and water extract (25.69 μg QE/mg) [10]. Similarly, successive extracts of another species of the same genus, i.e., *Dryopteris cochleata* leaves, were reported to contain considerable amounts of total phenolic contents, with 17.7 µg GAE/g petroleum ether, 32.9 µg GAE/g chloroform, 43.4 µg GAE/g ethyl acetate, 90.4 µg GAE/g acetone, 30.86 µg GAE/g methanol, and 28.4 µg GAE/g water. Furthermore, successive extracts of *Dryopteris cochleata* leaves were also found to contain a considerable amount of total flavonoid contents, with 9.16 µg catechin-equivalent (CE)/g petroleum ether, 122.5 µg CE/g chloroform, 145.78 µg CE/g ethyl acetate, 146.9 µg CE/g acetone, 77.71 µg CE/g methanol, and 25.74 µg CE/g water [11].

In the present study, radical scavenging potential was found to align with total phenolic and flavonoid contents. *D. juxtapostia* root dichloromethane extract exhibited considerable antioxidant potential in all three assays as compared to other extracts. These findings are supported by the fact that experimental variables, such as the type of solvent, are important with respect to estimation of antioxidant activity [35,36,37], as in the present study, dichloromethane extract was found to contain a considerable amount of phytochemicals, in addition to considerable radical-scavenging activity. These findings are in line with those reported by Kathirvel and Sujhata (2016) [11], i.e., that acetone extract of *Dryopteris cochleata* leaves exhibited notable radical-scavenging potential as compared to other tested extracts, owing to its total phenolic and flavonoid contents. Numerous reports have highlighted the antioxidant potential of plants due to the presence of phenolic compounds [38,39]. Additionally, it has been determined that the antioxidant activity of phenolics and flavonoids is mainly a result of their redox properties, which can play an important role in absorbing and neutralizing free radicals, quenching singlet and triplet oxygen, and decomposing peroxides Osawa et al. [40]. The radical-scavenging potential of *Dryopteris ramose* crude extract (91.95%), methanol fraction (88.25%), water fraction (87.28%), and ethyl acetate fraction (69.97%) was recently reported by Alam et al. (2021) [10] using a DPPH assay. *Dryopteris affinis*, another species from the same *genus* as DJ was reported to comprise a reasonable amount of total phenolic contents of 112.5 mg GAE/g. *Dryopteris affinis* rhizome extract showed antioxidant potential in DPPH (IC_50_ of 4.60 µg/mL) and ABTS (22.35 μmol Trolox/g) assays, which is even superior to that of standard butylated hydroxytoluene (BHT), with an IC_50_ of 9.96 µg/mL [41]. Another study reported the remarkable antioxidant potential of *Dryopteris ramose* dichloromethane (55.7% inhibition) and methanol extract (72.7% inhibition) in a DPPH assay parallel to standard quercetin (74.54% inhibition), further supporting the antioxidant potential of species belonging to this *genus.* A decade earlier, Kathirvel and Sujhata (2012) [42] reported that several extracts of *Dryopteris cochleata* leaves, i.e., acetone, ethyl acetate, methanol, chloroform, and water, exhibited reducing activities, with EC_50_ values of 243 µg, 327 µg, 378 µg, and 494 µg, respectively, in a dose-dependent manner. The current findings corroborate prior findings of antioxidant activity in components of the genus *Dryopteris*, although varying potency levels have been reported in the literature. Climate, geography, soil conditions, irrigation methods, harvesting timing, storage, transit facilities, drying procedures (shade drying, sun drying, oven drying, or freeze drying), the polarity of solvents, extraction methods, and extraction time could all play a significant role [19].

Urease is an enzyme that mediates the hydrolysis of urea, resulting in the production of ammonia and carbon dioxide, with its primary function being to protect bacteria in the acidic environment of the stomach [43]. Urease inhibitors have the potential to counteract urease’s detrimental effects on living organisms. Urease inhibitors are effective against a variety of infections caused by urease secretion by *Helicobacter pylori*, including gastrointestinal disorders, such as gastritis, duodenal ulcers, peptic ulcers, and stomach cancer [44]. Antibiotic treatment can heal ulcers, prevent recurrence of peptic ulcers, and reduce the risk of stomach cancer in high-risk groups. However, resistance to one or more antibiotics, as well as other considerations, such as poor patient compliance, drug side effects, and the considerable expense of combination therapy, have resulted in concerns among consumers with respect to safety, cost-effectiveness, and availability [45]. Figure shows 3 that *D. juxtapostia* root dichloromethane extract exhibited notable urease inhibition activity, followed by root methanol extract, exhibiting moderate urease inhibition activity. The results of the present investigation cannot be compared with previous data, as no species from this genus has previously be explored for antiurease activity, although various studies have reported the anti-inflammatory potential of this genus in other assays. For example, *Dryopteris crassirhizoma* ethanol extract was reported to diminish the mediation of nitric oxide and prostaglandin production in lipopolysaccharide-stimulated RAW264.7 cells. It also downregulated the levels of mRNA expression of pro-inflammatory genes, such as inducible nitric oxide synthase, cyclooxygenase, and TNF-α [46]. Some compounds isolated from water extract of *Dryopteris fragrans* were reported to have nitric oxide production inhibition potential in lipopolysaccharide-induced RAW 264.7 macrophages, with IC_50_ values of 45.8, 65.8, and 49.8 μM, respectively [47]. Another study explored *Dryopteris filixmas* leaves and reported that aqueous extract inhibited the hemolysis of red blood cell membranes (56.45% inhibition at 6 mg/mL) parallel to the inhibition induced by standard drug acetylsalicylic acid (70% inhibition at 6 mg/mL) [48]. These reports support the anti-inflammatory results of the present investigation, indicating that species from the genus *Dryopteris* possess health-promoting potential.

Carrageenan-induced paw edema is considered a credible anti-inflammatory compound screening test. The development of carrageenan-induced paw edema is a biphasic reaction, with the first phase including the release of kinins, histamine, and 5-HT and the second phase involving the release of prostaglandins [49]. Similarly, formaldehyde-induced pain and edema are mediated by bradykinins and substance P during the early phase of formaldehyde injection, whereas a tissue-mediated response in the later phase is associated with the release of histamine, 5-hydroxytryptamine (5-HT), prostaglandins, and bradykinins [50]. In our study, *D. juxtapostia* root dichloromethane extract was found to inhibit inflammation in carrageenan-induced (*p* < 0.001) and formaldehyde-induced (*p* < 0.001) paw edema models in a significant manner, suggesting that anti-inflammatory activity may be accredited to the inhibition of inflammatory mediators during both phases of edema formation. Recently, *S. cumini* extract was recorded to contain a considerable amount of total phenolic, flavonoid, and antioxidant potential, in addition to showing remarkable anti-inflammatory activities against carrageenan-, formalin-, and PGE2-induced intoxication [19]. Another group of researchers reported a direct relationship between phytochemical contents, antioxidant activity, and inhibition of inflammation [32], supporting the findings of current investigation. As previously stated, this is first report on the biological potential of *D. juxtapostia*, although many species of this genus have been found to offer inflammation aversion potential in the past. Ahmad et al. (2011) [12] reported that *Dryopteris chrysocoma* root extract exhibited significant (*p* < 0.001) inhibition of 51.1% when dispensed orally at a rate of 500 mg/kg after administration of formalin comparable to the standard aspirin. In the same study, significant inhibition (*p* < 0.001) of 57% was induced by *Dryopteris chrysocoma* root extract when administered at a rate of 500 mg/kg against carrageenan-induced paw edema, demonstrating the inflammation-averting potential of this genus. The inhibition was dose-dependent, and root extract was found to be more effective than leaf and stem extract. The findings are also in agreement with those reported by Khan et al. (2018) [13], i.e., that *Dryopteris blanfordii* extract showed significant analgesic activity (45% reduction in writhing, *p* < 0.01) when administered at a dose of 300 mg/kg, which is more than standard drug aspirin, exhibiting 40% inhibition at 150 mg/kg. In the same study, ethanolic extract of *Dryopteris blanfordii* exhibited significant (*p* < 0.01) inhibition against carrageenan-induced paw edema, with activity was parallel to the standard drug diclofenac sodium. Another species from the same genus, i.e., *Dryopteris crassirhizoma*, was reported to demonstrate significant antiallergic and anti-inflammatory activities by modulating the T-helper type 1 (Th1) and T-helper type 2 (Th2) response and reducing the allergic inflammatory reaction in phorbol myristate acetate (PMA)- and A23187-stimulated HMC-1 cells via NF-κB signaling in an ovalbumin (OVA)-induced allergic asthma model [51]. A decade earlier, radix methanolic extract of *Dryopteris crassirrhizoma* showed significant (*p* < 0.01) anti-inflammatory effects during the screening of almost 150 medicinal plants [52].

The findings of the brine shrimp assay revealed that *D. juxtapostia* root dichloromethane extract may contain some compounds that may have cytotoxic effects on certain cancer cells. As previously suggested by Mayer et al. (1982) [24], the brine shrimp lethality test can be used to predict compounds or extracts that may exhibit anticancer activity. These findings are in accordance with those reported by Baloch et al. (2019) [34], i.e., that dichloromethane extract of *Dryopteris ramosa* whole plant exhibited a very high potential for cytotoxicity, with an LD_50_ of 0.6903 µl/mL, which is 10 times more potent than etoposide, with an LD_50_ of 7.46 µl/mL. Another study reported *Dryopteris ramosa* to exhibit brine shrimp lethality potential, with an LD_50_ value of 47.64 μg/mL Alam et al. [10]. *Dryopteris affinis* rhizome and leaf methanol extracts were reported to exhibit moderate cytotoxicity in a brine shrimp assay, with LC_50_ values of 323.9 µg/mL and 85.5 µg/mL, respectively [41]. The methanol extract of *Dryopteris filixmas* leaves was also reported to exhibit potent cytotoxic activity, with an LC_50_ of 25.9 µg/mL, whereas the LC_50_ value of standard vincristine sulfate was 10.0 µg/mL [53].

*D.juxtapostia* root dichloromethane extract not only exhibited antioxidant, anti-inflammatory, and cytotoxic properties but also inhibited the proliferation HeLa human cervical and prostate cancer (PC3) cells (Table 6). Several species of the same genus have been reported to exhibit notable anticancer effects against different cancer cell lines [15,54,55], supporting the findings of the current investigation. The anticancer activity reported in the present study is consistent with total phenolics and flavonoids, anti-inflammatory effects, and cytotoxic potential. Anticancer activity of *Dryopteris cochleat* was previously believed to be associated with its phenolic and flavonoid contents [11,56]. *Dryopteris crassirhizoma* (50 and 100 g/mL) markedly inhibited the proliferation of PC-3 and PC3-MM2 cells without disturbing or inducing cytotoxicity toward normal spleen cells from BALB/C mice through the activation of caspase-3, -8, -9, bid, and PARP in PC3-MM2 cells [53].

## 5. Conclusions

The findings of the present investigation demonstrate the phytochemical, antioxidant, anti-inflammatory (in vitro and in vivo), and cytotoxic potential of *Dryopteris juxtapostia* (DJ) crude extracts, supporting the use of species belonging to this genus in traditional medicinal systems. DJ root dichloromethane extract exhibited the highest biological potential in all aforementioned assays, followed by DJ root methanol extract. Both extracts were found to exert no toxicity in the livers of the tested animals when administered at dosis 300 and 500 mg/kg according to liver function test, total protein, and lipid profile. Mass spectrometry analysis showed that some phenolic compounds are responsible for the antioxidant, anti-inflammatory, and anticancer potential of DJ. Overall, the current study confirms the potential of DJ with respect to bioactivity and as a possible alternative therapeutic vector. This research also provides a database for future research to optimize extraction methods and solvents for the maximum extraction of polyphenols and/or flavonoids. Furthermore, preserving the bioactivity of polyphenols and optimizing their delivery also represents a future challenge. In conclusion, plant polyphenols and flavonoids may represent a significant complementary medicine for treatment of oxidation- and inflammation-induced physiological dysfunction. However, further clinical trials are required to establish the safety and efficacy of bioactive compounds.

## Figures and Tables

**Table 1:** Hepatoprotective activity of DJ crude extracts.

Group No.	Treatment n = 4	Ten-Day Regimen	
		Days 1–7	Days 8–10
1	Normal saline (negative control)	Normal saline	Normal saline
2	Paracetamol (2 g/kg) (positive control)	Normal saline	Paracetamol (2 g/kg)
3	Silymarin (standard) (10 mg/kg)	10 mg/kg	Silymarin + Paracetamol
4	DJ DCM root (300 mg/kg)	300 mg/kg	DJ DCM root (300 mg/kg) + paracetamol
5	DJ DCM root (500 mg/kg)	500 mg/kg	DJ DCM root (500 mg/kg) + paracetamol
6	DJ MeOH root (300 mg/kg)	300 mg/kg	DJ MeOH root (300 mg/kg) + paracetamol
7	DJ MeOH root (500 mg/kg)	500 mg/kg	DJ MeOH root (500 mg/kg) + paracetamol
8	DJ DCM shoot (300 mg/kg)	300 mg/kg	DJ DCM shoot (300 mg/kg) + paracetamol
9	DJ DCM shoot (500 mg/kg)	500 mg/kg	DJ DCM shoot (500 mg/kg) + paracetamol
10	DJ MeOH shoot (300 mg/kg)	300 mg/kg	DJ MeOH shoot (300 mg/kg) + paracetamol
11	DJ MeOH shoot (500 mg/kg)	500 mg/kg	DJ MeOH shoot (500 mg/kg) + paracetamol

**Table 2:** Carrageenan-induced edema in rat hind paw.

Type of Extract	Dose mg/kg	0 h	1 h	2 h	3 h
		% Inhibition	% Inhibition	% Inhibition	% Inhibition
Control	-	-	-	-	-
Indomethacin	100	23.3 *	38.9 **	40.6 **	77.6 ****
DJ root DCM extract	200	16.2 ^ns^	18.5 ^ns^	39.5 **	61.7 ***
DJ root MeOH extract	200	17.0 ^ns^	22.8 *	28.8 *	43.9 **
DJ shoot DCM extract	200	9.30 ^ns^	14.2 ^ns^	20.4 *	24.4 *
DJ shoot MeOH extract	200	3.10 ^ns^	7.4 ^ns^	10.4 ^ns^	16.3 ^ns^

DJ, *Dryopteris juxtapostia*; DCM, dichloromethane; h, hours; MeOH, methanol; ns, non-significant. Values are presented as means ± S.D. of three measurements. * *p* < 0.05, ** *p* < 0.01, *** *p* < 0.001, **** *p* < 0.0001.

**Table 3:** Formaldehyde-induced edema in mouse hind paw.

Type of Extract	Dose mg/kg	1 h	3 h	6 h	12 h	24 h
		% Inhibition	% Inhibition	% Inhibition	% Inhibition	% Inhibition
**Control**	-	-	-	-	-	-
Indomethacin	100	68.7 ***	71.2 ***	76.6 ***	80.2 ***	86.3 ****
DJ root DCM extract	200	53.2 **	57.6 **	61.5 **	65.2 ***	67.3 ***
DJ root MeOH extract	200	29.9 *	28.7 *	34.5 *	44.9 **	45.1 **
DJ shoot DCM extract	200	15.5 ^ns^	19.2 ^ns^	23.5 ^ns^	25.5 *	31.9 *
DJ shoot MeOH extract	200	3.08 ^ns^	0.96 ^ns^	14.4 ^ns^	10.8 ^ns^	22.11 ^ns^

DJ, *Dryopteris juxtapostia*; DCM, dichloromethane; MeOH, methanol; ns. non-significant. Values are presented as means ± S.D. of three measurements. * *p* < 0.05, ** *p* < 0.01, *** *p* < 0.001, **** *p* < 0.0001.

**Table 4:** Toxicity assessment of various *D. juxtapostia* crude extracts using a brine shrimp lethality assay.

Extract	Dose (µg/mL)	% Mortality	EC_50_
DJ root DCM	1000	100 ± 0.00	26.74
	100	73.1 ± 2.45	
	10	10 ± 0.00	
DJ root MeOH	1000	20 ± 0.00	391.9
	100	3.3 ± 1.55	
	10	00 ± 0.00	
DJ shoot DCM	1000	49.9 ± 2.73	69.17
	100	20 ± 0.00	
	10	1.1 ± 1.55	
DJ shoot MeOH	1000	12.2 ± 1.55	208.8
	100	5.5 ± 1.55	
	10	00 ± 0.00	
Etoposide (standard drug)	10	71 ± 1.41	19.29

DJ, *Dryopteris juxtapostia*; DCM, dichloromethane; MeOH, methanol. Values are presented as means ± S.D. of three measurements.

**Table 5:** Cytotoxic activity of various *D. juxtapostia* crude extracts at 30 µg/mL determined by MTT assay.

Sample	Cell Line	% Inhibition	IC_50_ (µg/mL)
DJ root DCM	HeLa cervical cancer cell line	76.7 ± 0.5	17.1 ± 1.3
DJ root MeOH		62.9 ± 1.1	36.9 ± 0.9
DJ shoot DCM		34.4 ± 1.3	87.2 ± 1.1
DJ shoot MeOH		20.2 ± 0.9	143.6 ± 0.7
Doxorobicin (standard)		98.0 ± 1.1	0.90 ± 0.14
DJ root DCM	Human prostate cancer cell line	56.5 ± 0.1	45.2 ± 0.1
DJ root MeOH		30.6 ± 0.2	98.3 ± 1.1
DJ shoot DCM		28.5 ± 1.4	101.2 ± 2.1
DJ shoot MeOH		18.3 ± 0.6	187.4 ± 0.1
Doxorobicin (standard)		89.9 ± 0.12	1.90 ± 0.3

DJ, *Dryopteris juxtapostia*; DCM, dichloromethane; MeOH, Methanol. Values are presented as means ± S.D. of three measurements.

**Table 6:** Hepatoprotective analysis of *D. juxtapostia* root dichloromethane and methanol extracts.

Sample	Liver Function Test				Total Protein				Lipid Profile			
	Tb (mg/dL)	SGPT/ALT (IU/L)	SGOT/AST (IU/L)	ALP (IU/L)	SM	SA	Gb	A/G Ratio	Cholesterol (mg/dL)	Triglycerides (mg/dL)	HDL (mg/dL)	LDL (mg/dL)
Control	0.34	44	73.60	164	6.30	3.79	2.99	1.26	169	97	64	76
Paracetamol	0.8	103	151	497	7.96	3.22	4.03	0.97	243	152	53	95
Silymarin	0.49	66	104	241	7.16	3.62	3.01	1. 2	185	71	69	77
DJMR 300 mg	0.69	83	119	301	7.13	3.45	3.8	0.9	183	148	61	83
DJMR 500 mg	0.57	77	113	258	6.8	3.68	3.6	1.02	179	123	64	74
DJDR 300 mg	0.71	96	134	339	7.12	3.10	3.64	0.85	193	146	59	79
DJDR 500 mg	0.67	94	141	321	7.01	3.56	3.28	1.08	186	129	62	71

ALP, alkaline phosphate; SM, serum protein; SA, serum albumin; Gb, globulin; Tb, total bilirubin; SGPT/ALT, serum glutamic-pyruvic transaminase/alanine aminotransferase; SGOT/AST, serum glutamic-oxaloacetic transaminase/aspartate aminotransferase.

**Table 7:** ESI-MS/MS analysis of various crude extracts.

Sample Name	Compound	Average Mass (*m/z*)	ESI-MS/MS^n^ (*m/z*)	Mode	Identification	References
DJDR	**A**	433	432.3, 236.1, 196	Positive	Albaspidin PP	[30]
	**B**	445	445.2, 235.08, 223.08, 209.17	Negative	Flavaspidic acid BB	[31]
	**C**	627	625, 417.2, 403.1, 221,	Negative	Filixic acid ABP	[31]
	**D**	641	429, 417.2, 403.1, 237	Positive	Filixic acid ABB	[31]
	**E**	653	429, 417.2, 221.08	Positive	Tris-desaspidin BBB	[31]
	**F**	667	666.4, 442.3, 431, 235, 223	Positive	Tris-paraaspidin BBB	[31]
	**G**	863	866.4, 653.3, 625.3	Positive	Tetra-flavaspidic BBBB	[31]
	**G**	419	419, 223, 209, 196.9	Positive	Flavaspidic acid AB	[31]
	**I**	667	667.3, 653.4, 639.3, 431.1	Positive	Tetra-albaspidin BBBB	[31]
DJMR	**J**	290	289.1, 271.08, 247.08	Positive	Catechin	[19]
	**K**	291	273.1, 163.3, 139.08	Positive	Epi-catechin	[32]
	**L**	191	191, 173, 127	Positive	Quinic acid	[32]
	**M**	267	257, 237.1, 211.1,	Positive	Liquitrigenin	[33]

## Data Availability

The data is contained within the manuscript.
